# Imaging system QA of a medical accelerator, Novalis Tx, for IGRT per TG 142: our 1 year experience

**DOI:** 10.1120/jacmp.v13i4.3754

**Published:** 2012-07-05

**Authors:** Zheng Chang, James Bowsher, Jing Cai, Sua Yoo, Zhiheng Wang, Justus Adamson, Lei Ren, Fang‐Fang Yin

**Affiliations:** ^1^ Department of Radiation Oncology Duke University Durham North Carolina 27710 USA

**Keywords:** quality assurance, IGRT, TG 142

## Abstract

American Association of Physicists in Medicine (AAPM) task group (TG) 142 has recently published a report to update recommendations of the AAPM TG 40 report and add new recommendations concerning medical accelerators in the era of image‐guided radiation therapy (IGRT). The recommendations of AAPM TG 142 on IGRT are timely. In our institute, we established a comprehensive imaging QA program on a medical accelerator based on AAPM TG 142 and implemented it successfully. In this paper, we share our one‐year experience and performance evaluation of an OBI capable linear accelerator, Novalis Tx, per TG 142 guidelines.

PACS numbers: 87.57.‐s, 87.57.C‐, 87.57.uq, 87.59.‐e, 87.59.bd

## I. INTRODUCTION

Recent developments in image‐guidance enhance the accuracy of target localization and, as such, radiation can be delivered more precisely to the tumor while sparing adjacent healthy tissue. With the rapid development of imaging technology, image‐guided radiation therapy (IGRT) has been widely adapted into clinical practice. IGRT is often applied in many special hypofractionated treatments, such as stereotactic radiosurgery (SRS) and stereotactic body radiotherapy (SBRT).^(^ ^1^ ^–^ ^4^ ^)^ The utilization of any new technology and equipment necessitates a comprehensive quality assurance (QA) program to maintain and monitor system performance characteristics.^(^ ^5^ ^–^ ^10^ ^)^ The report of American Association of Physicists in Medicine (AAPM) Task Group (TG) 142 has been recently published and presents new recommendations for QA criteria of medical accelerators.^(^ ^5^ ^–^ ^6^ ^)^ More specifically, TG 142 is an update to AAPM TG 40,^(^ ^5^ ^)^ and has added recommendations for imaging devices that are integrated with the linear accelerator. The imaging devices include kilovoltage (KV) X‐ray imaging, megavoltage (MV) portal imaging, and cone‐beam CT (CBCT).

In this work, we share our one‐year experience of its implementation on a Novalis Tx system.

## II. MATERIALS AND METHODS

### A. Medical accelerator system

**Figure 1:**
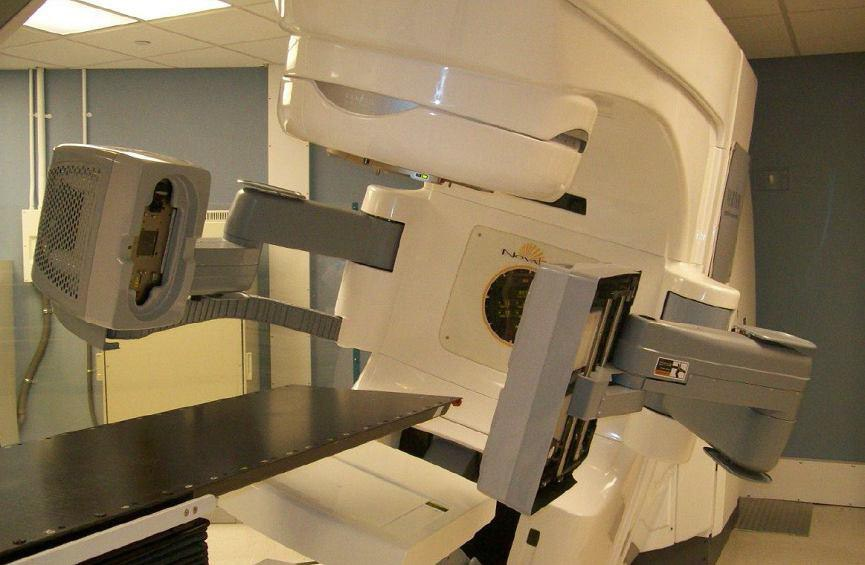
The Novalis Tx system equipped with MV EPID and KV OBI.

The Novalis Tx system (Varian, Palo Alto, CA, and BrainLAB, Heimstetten, Germany) is a megavoltage treatment unit modified based on a Varian Trilogy machine, as shown in Fig. 1. The Novalis Tx system is able to deliver high‐dose radiation through various modes. The system is directly integrated to the Varian 4DTC for treatment record and verification, MV electronic portal imaging device (EPID), and on‐board imager (OBI) applications using KV planar imaging and CBCT volumetric imaging.

#### A.1 Electronic portal imager device

The EPID used in the work is an amorphous silicon imaging device mounted on the linear accelerator. The image detector covers a total sensitive area of $40\times 30\;{\text{cm}}^{2}$ and features a matrix of 1024× 768 pixels with a resolution of $0.39\times 0.39\;{\text{mm}}^{2}$, which can be used for 2D radiographic acquisition or cine image acquisition.

#### A.2 On‐board imager for KV planar imaging and CBCT

As shown in Fig. 1, the OBI system consists of a kV X‐ray source (KVS) and a kV amorphous silicon detector (KVD) with a sensitive area of $40\times 30\;{\text{cm}}^{2}$. The OBI system provides three imaging modes: 2D radiographic acquisition, 2D fluoroscopic image acquisition, and 3D cone‐beam computed tomography (CBCT) acquisition. In CBCT, there are two acquisition modes available: “full‐fan” and “half‐fan”.^(^ ^7^ ^)^ To optimize CBCT imaging, a bowtie filter is placed in front of the kV beam to attenuate the edges of the kV beam. The bowtie filter reduces skin dose, avoids saturation of the detector, reduces X‐ray scatter, and reduces the effects of charge trapping in the detector.^(^ ^11^ ^)^

#### A.3 BrainLAB ExacTrac system

The system is also equipped with BrainLAB ExacTrac system, which includes a robotic couch, infrared (IR) and video detectors, and dual diagnostic kV X‐ray tubes and detectors. The two detectors are mounted on the ceiling, while the two X‐ray tubes are embedded on floors. In practice, the dual X‐ray devices are used to obtain a pair of oblique high‐quality radiographs. After a pair of images is acquired, the kV images are fused with the digitally reconstructed radiographs (DRR) obtained from simulation CT images. Once the desired fusion been reached, the couch parameters are transferred to the robotic couch, and the couch is moved to reposition patient with the guidance of the infrared system. The BrainLAB ExacTrac system is capable of taking intrafractional X‐ray images to monitor and drive three dimensional couch corrections for potential target movements between delivering beams.

### B. Equipments for imaging QA measurements

#### B.1 Las Vegas phantom

**Figure 2:**
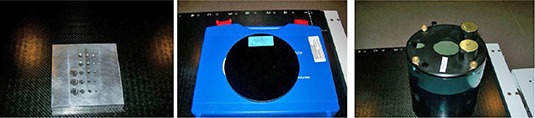
QA phantoms used to evaluate imaging systems: Las Vegas phantom (left), Leeds phantom (middle), Catphan 504 Phantom (right).

A Las Vegas phantom (Varian, CA) is used to evaluate image quality of EPID.^(^ ^12^ ^)^ As shown in Fig. 2, the Las Vegas phantom is a contrast‐detail aluminum phantom. The phantom has 28 circular holes with different depths and diameters. The spatial resolution and contrast of the MV EPID can be illustrated by the visibility of the holes, appearing as dark disks in a MV image.

#### B.2 Leeds phantom

A Leeds phantom TOR 18FG (Leeds Test Objects Ltd, North Yorkshire, UK) is used to quantify the spatial resolution and contrast of the planar KV OBI imaging, as shown in Fig. 2. The phantom has 18 disks of 8 mm diameter and 21 bar patterns.^(^ ^13^ ^)^ The spatial resolution and contrast can be specified by determining the lowest contrast disk and the smallest discernible group of bars visible in the image.

#### B.3 Catphan 504 phantom

A Catphan 504 Phantom (The Phantom Laboratory, NY) is used for the evaluation of the image quality of 3D CBCT, as shown in Fig. 2. The phantom has various inserts and, as a result, it can be used to measure different aspects of the CBCT image quality including geometric distortion, spatial resolution, low contrast, HU constancy, uniformity, and noise of CBCT.

#### B.4 Unfors Xi system

An Unfors Xi system (Unfors Instruments AB, Billdal, Sweden) is used to measure dosimetric characteristics of KV 2D OBI imaging and 3D CBCT. The Unfors Xi system consists of a base unit and several different external detectors. Different detectors are used to measure dosimetric characteristics of various modes, such as Radioscopy/Fluoroscopy (R/F) or Computed Tomography (CT). In this work, an R/F detector and a CT detector are used. The R/F detector is a solid‐state detector and has two sensors: R/F low and R/F high. R/F low is designed for low dose‐rate measurements with a phantom between the detector and the X‐ray source. R/F high is designed for conventional, high dose‐rate measurements, and is the sensor used in this work. The CT detector is a long cylindrical ionization chamber designed to measure CT dose for applications such as dose length product (DLP) and computed tomography dose index (CTDI).

### C. Imaging quality assurance for IGRT

Quality assurance (QA) is a critical component in a state‐of‐the‐art IGRT program. In this work, we focus on the procedures of the imaging QA program based on the AAPM TG 142 report and as demonstrated via successful implementation on a medical accelerator. These imaging quality assurance tests should be performed periodically to ensure the localization accuracy of the imaging system.

#### C.1 QA procedures of KV (OBI) and MV (EPID) planar imaging

##### C.1.1 Collision interlocks

Collision interlocks can be triggered by the interruption of motion of imaging devices and collision. The test was performed for MV detector (MVD), OBI KV source (KVS), and OBI KV detector (KVD). Also, users shall verify that alarm sounds and couch and gantry will not move when the interlock is triggered.

##### C.1.2 Mechanical QA

For on‐board imaging systems, the positioning accuracy of the X‐ray source and imaging panel should be checked periodically. More specifically, MVD, KVS, or KVD is first moved to a standard clinical position. Then, the lateral discrepancies and the distance from the isocenter to MVD, KVS, or KVD are measured with a ruler and compared with known reference distances.

##### C.1.3 Imaging positioning/repositioning QA

Positioning/repositioning accuracy is critical for the precise delivery of IGRT treatments. A phantom with small build‐in radiopaque targets can be used to perform the test (e.g., the cube phantom^(^ ^7^ ^)^. The phantom is first placed on the couch with the predefined target position at the isocenter. Then, the phantom is shifted away from the target position by known displacements along three translational directions. After this, the phantom is imaged and shifted back to the target position based on image matching. This test can be used to verify the accuracy of positioning/repositioning by the imaging systems.

##### C.1.4 QA of imaging & treatment coordinate coincidence

The test can be performed using a cube phantom, similar to positioning/repositioning accuracy QA. The phantom is first placed on the couch with the predefined target position at the isocenter. Planar MV and KV images are then taken at four cardinal gantry angles (e.g., 0°, 90°, 180°, and 270°). The distances between the corresponding digital imaging centers and the target center are measured as the discrepancies of the coincidence.

##### C.1.5 Scaling accuracy

**Figure 3:**
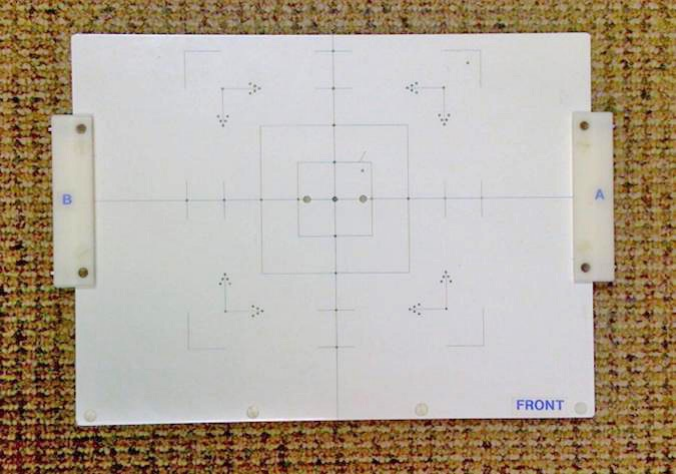
QA phantom used to perform the scaling accuracy test in our institute, in which multiple radiopaque markers are embedded.

The test can be performed by placing a blade calibration tool or an alternative adequate device at a standard clinical position (e.g., the isocenter at the neutral gantry angle). The QA device shall have at least five small radiopaque markers, which are arranged as follows: one marker is placed at the center, and the other four markers are placed peripherally from the central marker with a known distance in an orthogonal fashion. For example, Fig. 3 shows the picture of the calibration QA device used for the scaling accuracy test in our institute, in which multiple radiopaque markers are embedded. In practice, the QA device is first placed at the standard clinical position with the central marker aligned at the central axis. A pair of planar MV and KV images is then taken. The distance between the central marker and the peripheral markers can be measured and compared with the known distance.

##### C.1.6 Spatial resolution and contrast

###### C.1.6.1 MVD

**Figure 4:**
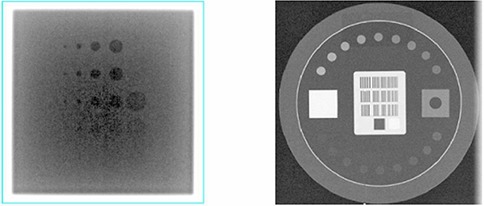
Planar images of imaging QA phantoms: the MV image of the Las Vegas phantom (left) and the KV image of the Leeds phantom (right).

The test can be performed by using the Las Vegas phantom. The Las Vegas phantom is placed on the top of couch and MVD is positioned at a stand clinical position (e.g., 50 cm away from isocenter along central axis). A planar MV image is acquired using 1 MU at a standard photon energy (e.g., 6 MV). As shown in Fig. 4, the Las Vegas phantom has 28 circular holes in a matrix of 6 columns (C) by 5 rows (R) embedded in the block. The maximum R visible in the maximum C tolerable determines contrast, and the maximum C visible in the maximum R tolerable determines resolution.

###### C.1.6.2 KVD

The test can be performed by using the Leeds phantom. The Leeds phantom is placed on the top of the KVD at a stand clinical position (e.g., 50 cm away from isocenter along central axis). In this work, a copper plate filter is placed on the KVS with a blade window size of 16 cm × 16 cm. A planar KV image is acquired at a predefined setting. As shown in Fig. 4, the number of visible disks determines the contrast. The number of bar‐pattern groups determines the resolution.

##### C.1.7 Uniformity and noise

When a uniform table top is available, a stack of 5 cm uniform plastic pieces of 30× 30 cm is placed on the uniform table top between the radiation source and the detector. A pair of planar MV and KV images is then taken at the most commonly used clinical settings. Image uniformity and noise can be quantified by postprocess analysis. More specifically, to measure image uniformity, a 1 cm × 1 cm square region of interest (ROI) is respectively placed at the image center and 7.5 cm off‐center left, right, top, bottom. The measured values of center, left, right, top, and bottom should agree with the average within ±5%. To quantify image noise, a 5 cm × 5 cm square ROI is placed at the center of the radiation field. The mean image intensity and the standard deviation (SD) of the intensity within the ROI are calculated. Fractional deviation (expressed as the ratio of SD/Mean) should agree with baseline values to be within 5%.

##### C.1.8 Full range of travel SDD (MVD)

The test can be carried out by placing the MVD at various positions covering the full range of travel (e.g., 5, 30, 50, and 70 cm). The distances between detector surface and isocenter are measured and compared against nominal values.

##### C.1.9 QA of beam quality and imaging dose

###### C.1.9.1 MV

The QA of beam quality and imaging dose for MV is generally performed during a periodical linear accelerator QA program. In general, the quality of an MV beam is characterized by the percent depth‐dose curve at a defined dosimetric condition, which can be verified periodically using a ratio of relative dose measurements at two different depths (e.g., 5 cm and 10 cm). Similarly, the imaging dose for MV can be tracked through a standard linear accelerator output calibration.

###### C.1.9.2 KV

**Figure 5:**
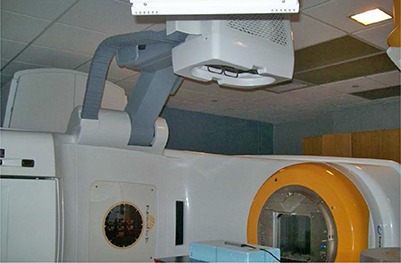
The QA of beam quality and imaging dose for KV is performed with the R/F high X‐ray detector placed on the top of blue styrofoam block.

The QA of beam quality and imaging dose for KV can be performed using the Unfors Xi system. As shown in Fig. 5, an R/F X‐ray detector is on the top of blue Styrofoam block placed at the position where the isocenter lies at the center of the detector. The longest dimension of the detector is aligned along with the in‐room saggital laser or cross‐hair along superior–inferior direction. X‐ray tube with the copper filter is placed at the anterior–posterior position. While KV images are acquired with the clinically used imaging protocols, the parameters of peak voltage (kVp) and imaging dose (mGy) are measured as the indicators of beam quality and dose for the corresponding KV protocols.

#### C.2 QA procedures of CBCT volumetric imaging

##### C.2.1 CBCT imaging & treatment coincidence and positioning/repositioning

In contrast to the QA of positioning/repositioning for planar imaging, a phantom with multiple built‐in small radiopaque targets is generally used to perform the test (e.g., the marker phantom^(^ ^7^ ^)^, which contains one target at the center and several peripheral targets. The phantom is first placed on the couch with the predefined target position, which is considered as “initial target position”. Then, CBCT is acquired and virtually shifted to match the small central and peripheral targets of the planning CT. The virtual shift is measured as the discrepancy of CBCT imaging and treatment coincidence. After this, the phantom is actually shifted away from the target position by known displacements along three translational directions. The phantom is scanned again with CBCT and is virtually shifted to match with the planning CT. The virtual shift is compared against the known displacement, and the difference is recorded as the discrepancy of CBCT volumetric image matching. After that, the virtual shifts are applied, and the couch is moved to a new couch position. Finally, the phantom is manually shifted back from the new couch position to the “initial target position”. The residual manual shift is recorded as the end‐to‐end discrepancy of positioning/repositioning of CBCT. In this test, three different discrepancies are measured and are used to verify the accuracy of CBCT.

##### C.2.2 QA of CBCT image quality

**Figure 6:**
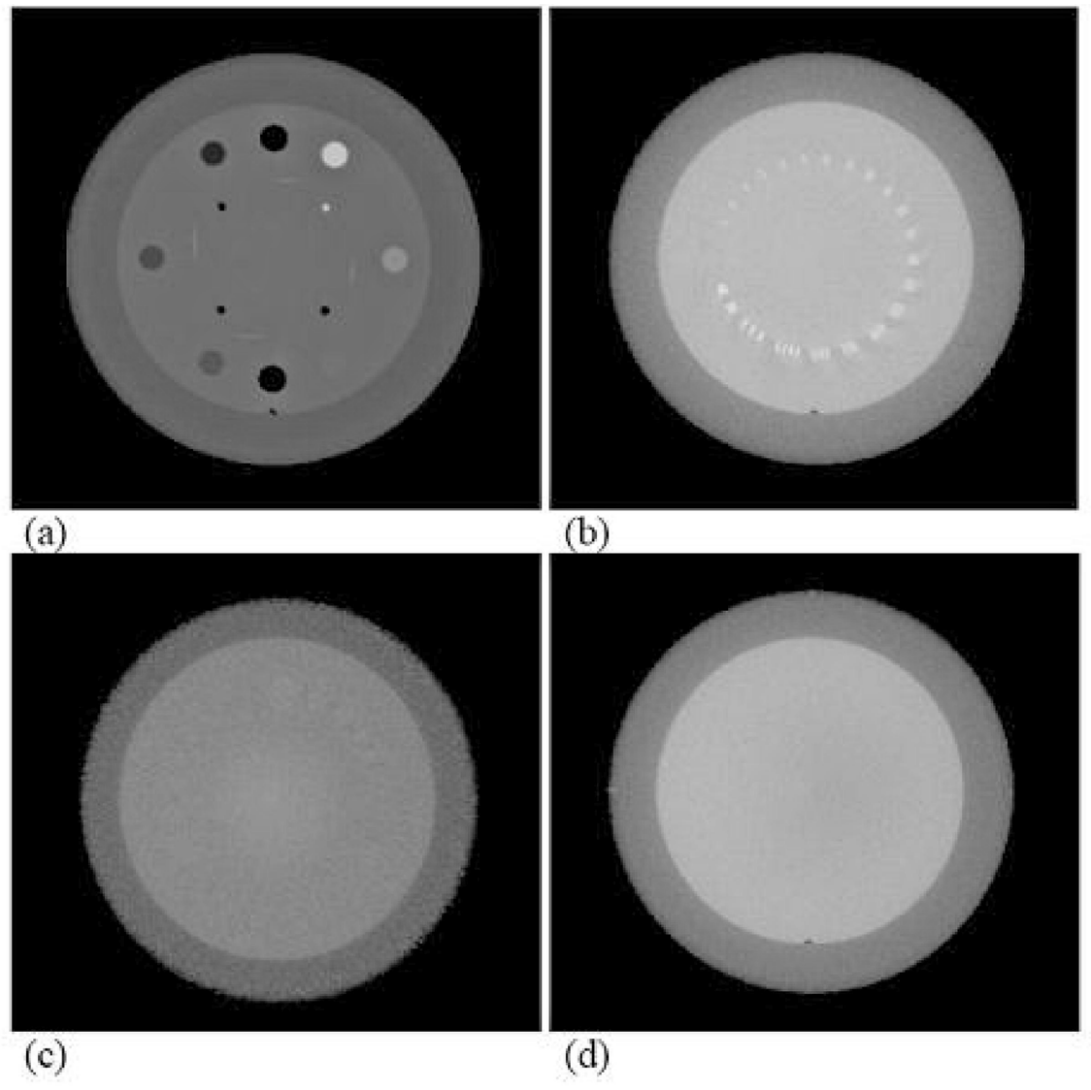
CBCT QA with a Catphan phantom: (a) Hounsfield unit verification; (b) high‐contrast resolution; (c) low‐contrast resolution; (d) Hounsfield unit uniformity and noise.

As shown in Fig. 6, the CBCT image quality QA can be performed with a standard CT phantom (e.g., the Catphan phantom), which includes geometric distortion, spatial resolution, low contrast resolution, Hounsfield unit (HU) constancy, uniformity, and noise. Since the methodology and procedures of these QA tests have been explicitly illustrated previously,^(^ ^7^ ^)^ they will not be discussed further in the work.

##### C.2.3 Imaging dose of CBCT

**Figure 7:**
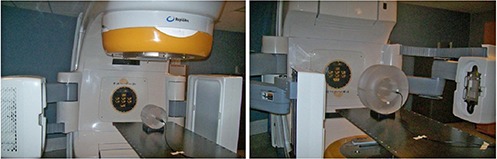
The QA of imaging dose for CBCT is performed with the CTDI chamber inserted at the center hole of the cylindrical phantom.

The imaging dose of CBCT can be measured using the Unfors Xi system with two cylindrical phantoms: one mimicking a human head and the other mimicking a human body. As shown in Fig. 7, a CTDI chamber is inserted at the center hole of each cylindrical phantom, where the isocenter lies at the center of the phantom. The chamber with the phantom is then scanned with CBCT. A dosimetric parameter termed integrated dose‐length value (IDLV) is measured as the indicator of dose for the corresponding CBCT mode. The parameter IDLV describes the integrated imaging dose over the length of 10 cm of the CT detector in the cylindrical phantom during the CBCT acquisition. The initial IDLV measurement is recorded and used as the baseline for the comparison with the future measurements of CBCT imaging dose.

The methodologies of these imaging quality assurance tests, along with the corresponding phantoms and measurement devices, have been discussed comprehensively. For convenience, a complete imaging QA form is included in this work as Appendix A.

## III. RESULTS

Comprehensive imaging QA tests of the imaging system of a medical accelerator for IGRT were performed on the Novalis Tx System. This work presents the QA results of a one‐year period, for future reference and comparison.

### A. QA of KV (OBI) and MV (EPID) planar imaging

#### A.1 Collision interlocks

The safety feature has been tested daily. On rare occasions, the safety tests did not pass and calibration files are lost, requiring immediate correction. The calibration of the detector/source is lost sometimes when the motion of the detector/source comes to an abrupt stop while in motion. To avoid calibration files being lost, the test can be performed while the detector/source is stationary.

#### A.2 Mechanical QA

For MV (EPID) and KV (OBI) imaging systems, the positioning accuracy was checked monthly.

##### A.2.1 MVD

**Figure 8:**
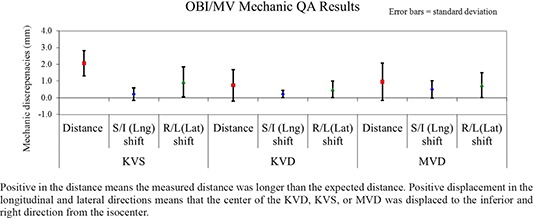
Positive in the distance means the measured distance was longer than the expected distance. Positive displacement in the longitudinal and lateral directions means that the center of the KVD, KVS, or MVD was displaced to the inferior and right direction from the isocenter. Results of mechanic QA for KVD, KVS, and MVD.

The MVD‐to‐isocenter distance was measured with a ruler and compared with known reference distances. In the test, the MVD with the cover is moved to the position of 50 cm away from isocenter along the central axis. The distance was measured within 2 mm from the reference value of 46.8 mm, and the lateral discrepancy was also measured within 2 mm over a period of one year. The results were illustrated in Fig. 8.

##### A.2.2 KVS and KVD

The OBI KVS‐to‐isocenter distance and isocenter‐to‐KVD distance were measured with a ruler and were compared with known reference distances. The average measured distance displacement was 2.1±0.8mm for source‐to‐isocenter distance, 0.2±0.4mm for the source shift along longitudinal direction, and 1.0±0.9mm for the source shift along lateral direction. In contrast, the average measured displacement was 0.7±0.9mm for isocenter‐to‐detector distance, 0.2±0.2mm for the detector shift along longitudinal direction, and 0.5±0.5mm the detector shift along lateral direction. The measured discrepancies were all within 2 mm. The results were summarized in Fig. 8.

#### A.3 Imaging positioning/repositioning QA

A cube phantom with one small radiopaque target was used to perform the test. Specifically, the phantom was first placed on the couch with the built‐in target at the isocenter. Then, the phantom was shifted away from the target position by 1.0 cm along longitudinal, lateral, and vertical directions. After this, the phantom was imaged and shifted back to the target position based on image matching. The discrepancies were measured within 2 mm from the reference location over a period of one year.

#### A.4 QA of imaging & treatment coordinate coincidence

##### A.4.1 MV PV imaging isocenter accuracy

**Figure 9:**
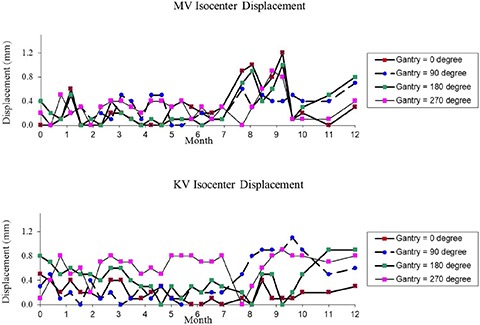
Results of the 29 measurements of the QA of Imaging & Treatment Coordinate Coincidence over a period of 12 months: MV PV imaging isocenter accuracy (top) and KV OBI imaging isocenter accuracy (bottom). The vector displacement was measured between the center of the digital graticule and the center marker in the image.

The accuracy of the MV PV imaging isocenter was verified by using a cube phantom with a small target ball at the center over a 12‐month period, which was illustrated in Fig. 9. For example, the average measured imaging isocenter displacement of MV PV device was 0.39±0.34mm for anterior–posterior (AP) setup position at gantry=0°, and 0.40±0.32mm for right‐lateral (RLAT) setup position at gantry =270°. Starting at the seventh month, imaging isocenter displacement approached the tolerance level of 1 mm. Clinical engineers have helped adjust the imaging isocenter by multiple calibrations. During that period, one measurement exceeded 1 mm and occurred at AP setup with a maximum displacement of 1.3 mm. The following repeated measurement was 0.5 mm after the cube phantom was recalibrated.

##### A.4.2 KV OBI imaging isocenter accuracy

The accuracy of the KV OBI imaging isocenter was also verified over the one year of measurements, in a similar fashion to MV PV isocenter verification, which was illustrated in Fig. 9. The average measured imaging isocenter displacement of KV OBI was 0.39±0.34mm for AP setup position at gantry =90°, and 0.75±0.23mm for RLAT setup position at gantry =0°. Due to multiple calibrations, discrepancies of the KV OBI imaging isocenter varied considerably, starting in the seventh month, as illustrated in Fig. 9. Compared with the OBI results presented in the previously published data,^(^ ^7^ ^)^ the OBI isocenter accuracy shows improved performance on the Novalis Tx System.

#### A.5 Scaling accuracy

The test was performed by using a calibration tool, as shown in Fig. 3. A pair of planar MV and KV images was taken to verify the scaling accuracy. The discrepancies of the measured distances between the central marker and the peripheral markers were within 1 mm over one year.

#### A.6 Spatial resolution and contrast

##### A.6.1 MVD

The test has been performed by using the Las Vegas phantom. The maximum R visible greater than R2 in the maximum C tolerable greater than C5 was generally maintained for the contrast. The maximum C visible greater than C3 in the maximum R tolerable greater than C3 was generally maintained for the resolution.

##### A.6.2 KVD

The test has been performed by using the Leeds phantom, while a copper plate filter was placed on the KVS with a blade window size of 16 cm × 16 cm. A planar KV image is acquired at the predefined setting (70 kVp, 32 mA, and 6 ms). The number of visible disks was 11 or greater, and the number of visible bar‐pattern groups was 10 or greater, indicating a resolution of 1.40 lp/mm or better.^(^ ^13^ ^)^

#### A.7 Uniformity and noise

Planar anterior–posterior (AP) MV and KV images were both taken with a stack of 5 cm solid water phantom with the most commonly used clinical settings. Specifically, the MV image was acquired with a field size 20 cm × 20 cm at the energy of 6 MV, while the detector was placed at SDD=150 cm and couch was moved to set SSD=100 cm to plastic surface. The KV image was taken at the clinical setting (100 kVp, 200 mA, 40 ms), while the detector was placed at SDD=150 cm. Image uniformity and noise were measured to agree with baseline values to within 1% over the period of 12 months.

#### A.8 Full range of travel SDD (MVD)

The test was carried out by placing the MVD at three positions (i.e., 30, 50, and 70 cm). The distances between detector surface and isocenter were measured and agreed with nominal values within 3 mm.

#### A.9 QA of beam quality and imaging dose

##### A.9.1 MV

The imaging dose for MV was maintained within 2% from the nominal value through monthly output calibration over one year.

##### A.9.2 KV

The QA of beam quality and imaging dose for KV has been performed using the Unfors Xi system. As illustrated in Table 1, the parameters of peak voltage (kVp) and imaging dose (mGy) were measured as the indicators of beam quality and dose for the corresponding KV protocols. Only a copper filter (no bowtie) was used for kV imaging dose measurement. We noticed differences between the peak voltage and the corresponding measured values for the Extremity protocol. However, repeated measurements have yielded consistent results.

**Table 1:** Baseline measurements of the parameters of peak voltage (kVp) and imaging dose of planar KV imaging using the Unfors Xi system.

*KV OBI Protocols*	*Peak Voltage (kVp)*	*Peak Voltage (kVp) Baseline*	*Imaging Dose (mGy) Baseline*
Pelvis‐AP‐Med	75	81.56	0.03
Pelvis‐Lat‐Med	105	103.9	1.07
Pelvis‐AP‐Large	75	80.66	0.04
Pelvis‐Lat‐Large	120	119.4	2.83
Head‐AP	100	98.76	0.08
Head‐Lat	70	81.09	0.01
Thorax‐AP	75	85.10	0.01
Thorax‐Lat	95	92.67	0.35
Abdomen‐AP	80	83.96	0.12
Abdomen‐Lat	85	87.25	0.21
Extremity	65	83.56	0.003

### B. QA procedures of CBCT volumetric imaging

#### B.1 CBCT imaging & treatment coincidence and positioning/repositioning

A marker phantom was used to verify the coincidence of CBCT imaging and treatment isocenters, and CBCT positioning/repositioning. The marker phantom is shaped as a solid block with one fiducial marker at the center and four markers at known locations inside the phantom. The average measured displacement of CBCT positioning/repositioning was 0.3±0.4mm, 0.0±0.0mm, and 0.0±0.0mm in vertical, longitudinal, and lateral directions, respectively. The measured discrepancies of the coincidence of CBCT imaging and treatment isocenters were within 1.0 mm over the period of 12 months.

#### B.2 QA of CBCT image quality

The CBCT image quality test has been performed monthly with a Catphan phantom.

i) *CBCT Hounsfield unit verification:* Table 2 shows the average HU values along with the standard deviation of the results for the HU verification over the period of 12 months. The average discrepancies in HU of seven different materials were well within 40 HU.
ii) *CBCT slice thickness:* The measured displacement was within 0.5 mm. For example, the average measured displacement of slice thickness was −0.1±0.3mm for a full‐fan standard‐dose head mode, and –0.1±0.3mm for a half‐fan pelvis mode.
iii) *CBCT spatial linearity:* The discrepancy was well within 1 mm over the measurements of the 12 months. For example, the average measured distance deviation was 0.1±0.2mm for a full‐fan standard‐dose head mode, and −0.3±0.3mm for a half‐fan pelvis mode.
iv) *CBCT high‐contrast resolution:* Generally, the bars in Group #6 or better were seen on the Catphan phantom in CBCT. The Group #6 corresponds to a spatial resolution of 6 line/cm with a gap size of 0.083 cm. Since the results are subjective, the test is simply to verify the constancy of imaging performance.
v) *CBCT low‐contrast resolution:* 1.0% contrast or better of the Catphan phantom were visible on the Catphan phantom in CBCT. Similar to high‐contrast resolution test, this test is also subjective, and is used to ensure consistent imaging characteristics over time.
vi) *CBCT uniformity:* The HU uniformity was generally within ±40 HU over the measurements of one year. For example, over one year, the average measured HU was 9±14 in ROI at the center, 14±17 in ROI at top, 8±11 in ROI at bottom, 5±14 in right ROI, and 22±13 in left ROI in a full‐fan standard‐dose head mode. Similarly, the average measured HU was 10±17 in ROI at the center, 10±14 in ROI at top, 7±15 in ROI at bottom, 5±20 in right ROI, and 11±17 in left ROI in a half‐fan pelvis mode. The data are summarized in Table 3 for easy comparison. Compared with the CBCT image quality data presented in the previously published work,^(^ ^7^ ^)^ the CBCT imaging system shows consistent performance on the Novalis Tx System.

**Table 2:** The average HU values along with the standard deviation (SD) of the results for the HU verification over the period of 12 months.

*Phantom Materials*	*Expected HU*	*Full‐Fan Av. Measured HU*±*SD*	*Half‐Fan Av. Measured HU*±*SD*
Air	−1000	−986±13	−993±11
PMP	−200	−198±14	−192±10
LDPE	−100	−105±15	−97±6
Polystyrene	−35	−34±6	−33±11
Acrylic	120	122±14	122±7
Delrin	340	342±7	348±6
Teflon	990	998±30	1006±26

**Table 3:** The average HU values along with the standard deviation (SD) of the results for the CBCT HU uniformity measurement over the period of 12 months.

*Location of ROI*	*Full‐Fan Av. Measured HU*±*SD*	*Half‐Fan Av. Measured HU*±*SD*
Center	9±14	10±17
Top	14±17	10±14
Bottom	8±11	7±15
Right	5±14	5±20
Left	22±13	11±17

#### B.3 Imaging dose of CBCT

The imaging dose of CBCT has been measured using the Unfors Xi system with a cylindrical phantom mimicking a human body. As illustrated in Table 4, the IDLV measurements were acquired as the indicator of dose for the corresponding CBCT mode. The initial IDLV measurements were used as the baseline.

**Table 4:** Baseline measurements of the imaging dose of CBCT using the Unfors Xi system with a cylindrical phantom mimicking a human body.

*CBCT Protocols*	*Phantom*	*Integrated Dose‐length Value Baseline (mGy.cm)*
Standard‐dose Head	Small	50.9
Low‐dose Head	Small	26.3
High‐quality Head	Small	250.5
Pelvis	Large	139.4
Pelvis Spot Light	Large	158.1
Low‐dose Thorax	Large	37.1

## IV. DISCUSSION

In this work, we share our one‐year experience in the development of a comprehensive imaging QA program based on TG 142 recommendations to monitor accuracy of the imaging system for IGRT, and we performed systematic measurements for various imaging QA tests on a Novalis Tx linear accelerator. The QA tests cover imaging system dosimetry, imaging quality, mechanical accuracy, and geometrical accuracy. In practice, it usually takes 3–5 hours per month for a physicist to complete all the QA tests. Since annual QA merely adds consistency of radiation dose, it adds only marginal extra time to a monthly QA. The time may vary depending upon the proficiency of the physicist. There are many commercial software now available (e.g., DoseLab Pro, RIT) that may reduce this time still further; however, we did not test any of them.

In this work, a marker phantom was demonstrated in the QA procedures of CBCT imaging and treatment coincidence and CBCT positioning/repositioning. In the cases where a treatment of stereotactic radiosurgery is localized using CBCT, the standard Winston‐Lutz test phantom can be used for the CBCT QA test. The standard Winston‐Lutz test phantom shall contain a single target ball that is used to verify accuracy of radiation isocenter. In this case, the “initial target position” or the reference target position shall be the position where a Winston‐Lutz test is passed. Therefore, the QA test needs to be performed right after the Winston‐Lutz test.

As demonstrated from the measurements at our institute, the accuracies and consistencies of the data are acceptable when adequate QA devices and phantoms are used. For example, the average accuracy of the OBI isocenter was generally better than 1.0 mm with a standard deviation of less than 0.5 mm. The positioning/repositioning accuracy was within 1.0 mm or better along three directions. These results are not only valuable in ensuring accuracy of the imaging system, but also provide solid data to help prescribe adequate margins for clinical treatments, where imaging guidance is used for localization.

As described previously, the Novalis Tx system is also equipped with BrainLAB ExacTrac system, which includes dual diagnostic kV X‐ray tubes and detectors. To ensure its accuracy, the system is recommended to be calibrated every day on X‐ray isocenter and X‐ray quality using the dedicated BrainLAB isocenter calibration phantom and X‐ray calibration phantom, respectively. For SRS and SBRT procedures, IGRT QA for this system is recommended to meet tighter daily QA tolerances of ≤1 mm for the imaging and treatment coordinate coincidence, and the accuracy of positioning/repositioning per TG 142. These have been verified daily during the 12‐month period of QA experience with the system. In fact, the calibration was conducted on a daily basis at our institute. The X‐ray quality calibration was successfully performed and passed every day, and the isocenter verification tests showed the discrepancies well within 1 mm daily. Details of the QA phantom and ExacTrac calibration procedures can be found in ExacTrac user manuals,^(^ ^14^ ^)^ but will not be explicitly discussed in this work.

As image guidance has emerged in radiotherapy, the radiation exposure from imaging should be carefully managed in clinical practice. To fulfill clinical needs, AAPM TG 142 recommends that annual imaging dose measurements shall be performed and compared to acquired baseline data. Also, AAPM Task Group 75 has recently published a report on the management of imaging dose during image‐guided radiotherapy.^(^ ^15^ ^)^ This report reviews image‐guidance techniques along with their radiation doses, and recommends optimization strategies to trade off imaging dose with improvements in radiation delivery. Although imaging systems reviewed in the report are not exactly as those in this work, dose measurements of similar imaging systems in both works show generally consistent results. For example, the radiographic doses reported in TG 75 report range from 0.1 to 2.0 mGy for KV planar radiographic imaging; similarly, the radiographic doses are reported from 0.01 to 2.83 mGy for KV planar radiographic imaging in this work.

In the AAPM TG 142 report, various imaging QA tests are recommended, along with the corresponding criteria and frequencies. In this work, our QA data were acquired according to the recommended frequencies or even with greater frequencies. For example, the QA test of imaging and treatment coordinate coincidence for MV/KV planar imaging was generally performed bi‐weekly, with a greater frequency than that recommended by TG 142. The measurements over 12 months demonstrate that isocenters of the MV/KV imaging systems on a Novalis TX system are stable, and suggest that a monthly measurement for the QA test shall be adequate. Similarly, the CBCT quality test data demonstrate stable characteristics of CBCT over one year. The data might suggest that full‐fan or half‐fan mode can be alternated when tested every month, with considerable reduction of QA time. In the AAPM TG 142 report, dosimetric imaging QA is recommended annually. It is, however, important that the QA tests shall be performed at the time of commissioning or after major repairs, to establish a baseline and afterwards on a regular basis as suggested by TG 142 to have necessary confidence in the accuracy of the system.

## V. CONCLUSIONS

Our one‐year evaluation of a Novalis Tx linear accelerator demonstrates excellent compliance with TG 142 guidelines, thereby giving confidence in the quality of treatment being administered to the patients. The TG 142 compliance may require additional hardware in the form of special phantoms and may take 3–5 hours of a physicist's time per linear accelerator per month.

## Imaging Quality Assurance Procedures for IGRT

Physicist:____________________________Date:______________

**Table:**

	*TREATMENT IMAGING TEST*	*TOLERANCE*	*Finding*	*Initial*
	**kV and MV (EPID) Imaging**			
I1	Collision interlocks	Functional		
I2	Mechanic QA	≤2 mm		
I3	Positioning/repositioning	≤2 mm for non‐SRS/SBRT ≤1 mm for SRS/SBRT		
I4	Imaging & Treatment coordinate coincidence (four gantry angles)	≤2 mm for non‐SRS/SBRT ≤1 mm for SRS/SBRT		
I5	Scaling	≤2 mm for non‐SRS/SBRT ≤1 mm for SRS/SBRT		
I6	Spatial resolution	Baseline		
I7	Contrast	Baseline		
I8	Uniformity and noise	Baseline		
I9	Full range of travel SDD (MV imaging) (Annual QA only)	±5 mm		
I10	Beam quality / energy (kV imaging) (Annual QA only)	Baseline		
I11	Imaging dose (Annual QA only)	Baseline		
	**Cone‐beam CT (kV)**			
I12	Imaging & treatment coordinate coincidence ≤1 mm for SRS/SBRT	≤2 mm for non‐SRS/SBRT		
I13	Positioning/repositioning ≤1 mm for SRS/SBRT	≤2 mm for non‐SRS/SBRT		
I14	Geometric distortion	≤ 1 mm		
I15	Spatial resolution	Baseline		
I16	Contrast	Baseline		
I17	HU constancy	Baseline		
I18	Uniformity and noise	Baseline		
I19	Imaging dose (Annual QA only)	Baseline		

## I1. Collision Interlocks

Date:_____ Initial:_____

Collision interlocks can be triggered by the interruption of motion of imaging devices and collision. The test was performed for MV detector, OBI KV source, and OBI KV detector. More specifically, while imager is in motion, press imager and verify that imager stops. Verify that alarm sounds and couch and gantry will not move, when the interlock is triggered.

**Table:**

		*MVD*	*KVS*	*KVD*
Arm pedal	Alarm sound	□	□	□
Device body	Alarm sound	□	□	□
Motion disabled	Arm motion	□	□	□
	Gantry motion	□	□	□
	Couch motion	□	□	□

**Pass/Fail:** _____

## I2. Mechanical QA

Date: _____ Initial:_____

### MVD

Set the MVD at (0, 0, 50). (Leave the cover on the MVD.) Measure the distance between the detector‐cover surface and isocenter.

**Table:**

*Reading (cm)*	*Distance*	*S/I (Lng) Shift*	*R/L (Lat) Shift*
Expected Value (cm)	46.8±0.2	≤0.2	≤0.2

### KVS

Set the KVS at (0, 0, 100) and gantry at 270° so that the KVS is on the floor. Measure the distance between the KVS metal plate and the isocenter. Read the shift between the mark on the plate and the lasers projected from the ceiling.

**Table:**

*Reading (cm)*	*Distance*	*S/I (Lng) Shift*	*R/L (Lat) Shift*
Expected Value (cm)	85.2±0.2	≤0.2	≤0.2

### KVD

Measure from the cover of KVD. Set the KVD at (0, 0, 50) and gantry at 90° so that the KVD is on the floor. Measure the distance between the KVD grid surface and the isocenter. Read the shift between the mark and the lasers projected from the ceiling.

**Table:**

*Reading (cm)*	*Distance*	*S/I (Lng) Shift*	*R/L (Lat) Shift*
Expected Value (cm)	45.2±0.2	≤0.2	≤0.2

**Pass/Fail:** _____

## I3. Positioning/repositioning

Date: _____ Initial:_____

“2D2D Match” → “Analyze” → “Manual Match” → Move the blend bar to the right so that DRR disappears → Grab the red graticule to match the center of the red graticule to the off‐axis BBs. → “Apply shift” → Enable motion in Clinac → “Done” → Clear mode up in 4DiTC. → Retract MVD/KVD/KVS from the console → Go into the room, and align the off‐axis BBs to the isoceneter per crosshair and lasers.

### a. MV

**Table:**

	*Shifts (cm)*	*New Couch Position (A)*	*Final Couch Position (B)*	□*A*‐*B*□	*Results*
Couch Vert.					OK ≤2 mm
Couch Lng.					OK ≤2 mm
Couch Lat.					OK ≤2 mm

### b. KV

**Table:**

	*Shifts (cm)*	*New Couch Position (A)*	*Final Couch Position (B)*	□*A*‐*B*□	*Results*
Couch Vert.					OK ≤2 mm
Couch Lng.					OK ≤ 2 mm
Couch Lat.					OK ≤ 2 mm

**Pass/Fail:**_____

## I4. Isocenter consistency per Gantry Rotation

Date:_____Initial:_____

Mode up each field and take an image. Mark the BB and measure the distance between the graticule center and the BB center.

**Pass/Fail: ______**

## I5. Scaling Accuracy

Date:_____ Initial:_____

Place the blade calibration tool or alternative adequate device at 100SSD. Take MV and KV images. Place the digital graticule. Measure the distance between the center BB and the graticule center and the vertical and horizontal distances between the peripheral BBs.

### a. MV

**Table:**

*Reading (cm)*	*S/I (Lng) Shift*	*R/L (Lat) Shift*	*Vertical Line*	*Horizontal Line*
Expected Value (cm)	≤0.2	≤0.2	10.0±0.2	10.0±0.2

### b. KV

**Table:**

*Reading (cm)*	*S/I (Lng) Shift*	*R/L (Lat) Shift*	*Vertical Line*	*Horizontal Line*
Expected Value (cm)	≤0.2	≤0.2	10.0±0.2	10.0±0.2

**Pass/Fail:**_____

## I6‐I7. Spatial Resolution and contrast

Date:_____Initial:_____

### a. MVD

Set MVD at (0, 0, 50) and place the Vegas phantom on the top of couch. Acquire an image using one the appropriate field in OBI, QA. (This field should set the jaws tightly about the phantom.) Deliver 1 MU at 6 MV. The maximum R visible in C1 determines contrast, and the maximum C visible in R1 determines resolution.

Row visible:________________(Tolerance: ≥R4)

Row tolerance:________________ (Tolerance: ≥C3)

Column visible:________________ (Tolerance: ≥C5)

Column tolerance:________________ (Tolerance: ≥R2)

### b. KVD

Set KVD at (0, 0, 50) and place the Leeds phantom on the top of the KVD. Stick the copper plate on the KVS window. Acquire an image with 16 cm × 16 cm blade size, fluoro, 70 kVp, 32 mA, and 6 ms. The number of visible disks determines the contrast.

Leave the copper plate. Acquire an image with 16 cm × 16 cm blade size, fluoro, 75 kVp, 80 mA, and 32 ms. Select the clearly visible group of 41/2 line pairs. Measure the distance (A) of from the first line to the last line. The resolution is 4.5 line pairs/A.

Contrast:___________disks (> 11 to 12 disks)

Resolution:______________ lp/ mm (≥1.25 lp/mm or group 9)

### c. MVD Cassette Calibration

Cassette calibration performed? Yes / No (circle one).

Low dose image: 6X 400MU/min, 6X 100MU/min, 15X 400MU/min, 15X 100MU/min

High quality: 6X 400MU/min, 6X 100MU/min, 15X 400MU/min, 15X 100MU/min

Integrated Image: 6X 300MU/min, 6X 400MU/min, 6X 500MU/min, 6X 600MU/min, 15X 300MU/min, 15X 400MU/min, 15X 500MU/min, 15X 600MU/min

Continuous mode: 6X 300MU/min, 6X 400MU/min, 6X 500MU/min, 6X 600MU/min, 15X 300MU/min, 15X 400MU/min, 15X 500MU/min, 15X 600MU/min

**Pass/Fail:**_____

## I8. Uniformity and Noise

Date:_____Initial:_____

Use a uniform table top and add 5 cm of uniform plastic pieces of 30× 30cm between the radiation source and the detector. Other parameters: MV: field size 20× 20, 6 MV, detector at 150 cm, 100 SSD to plastic surface, gantry angle =0°. Take an image under the low dose mode; OBI: the most commonly protocol used on the corresponding machine (e.g., head AP, KVp=100, mA=200, mS=40).

Image Uniformity analysis: Place a 1 cm by 1 cm square ROI (region of interest) at the image center and 7.5 cm off‐center left, right, top, bottom. The measured values of center, left, right, top, and bottom should agree with the average to within ±5%.

Image Noise analysis: draw a 5 cm by 5 cm square ROI at the center of the radiation field. Measure the average image intensity and the SD (standard deviation) of the intensity within the ROI. Fractional deviation (SD/Mean) should agree with baseline values to within 5%.

### a. MV

**Table:**

	*Shifts (cm)*	*New Couch Position (A)*	*Final Couch Position (B)*	□*A*‐*B*□	*Results*
Couch Vert.					OK ≤2 mm
Couch Lng.					OK ≤2 mm
Couch Lat.					OK ≤2 mm

### b. KV

**Table:**

	*Shifts (cm)*	*New Couch Position (A)*	*Final Couch Position (B)*	□*A*‐*B*□	*Results*
Couch Vert.					OK ≤2 mm
Couch Lng.					OK ≤2 mm
Couch Lat.					OK ≤2 mm

**Pass/Fail:**_____

## I9. Full Range of Travel sdd (MVD) (Annual QA only)

Date:_____ Initial:_____

Set the MVD at various positions covering the full range of travel (e.g., 5, 30, 50, and 70 cm). Measure distance between detector surface and isocenter with a tolerance of 5 mm.

**Pass/Fail:**_____

## I10–I11. Image dose (Annual QA only)

Date:_____Initial:_____

### a. MV

The amount of MU used for taking the images is a good indicator of imaging dose. The therapist staff should be reminded to minimize MU for imaging purpose.

### b. KV

Devices: R/F High X‐ray detector, copper plate filter, and blue Styrofoam block.

Setup condition: R/F High X‐ray detector is on the top of blue Styrofoam block placed at the position where the isocenter lies at the center of the detector. The longest dimension of the detector is aligned along with H‐F laser or crosshair. X‐ray tube with copper filter is placed at AP position.

**Table:**

*KV OBI Protocols*	*Peak Voltage (kVp)*	*Peak Voltage (kVp) Baseline*	*Year*	*Imaging dose (mGy) Baseline*	*Year*
Pelvis‐AP‐Med	75				
Pelvis‐Lat‐Med	105				
Pelvis‐AP‐Large	75				
Pelvis‐Lat‐Large	120				
Head‐AP	100				
Head‐Lat	70				
Thorax‐AP	75				
Thorax‐Lat	95				
Abdomen‐AP	80				
Abdomen‐Lat	85				
Extremity	65				

**Pass/Fail:**_____

## I12–I13. cBcT Imaging & Treatment coincidence & Positioning/repositioning

Date:_____Initial:_____

A. Set the marker block phantom based on the crosshair and laser. Record the couch position.
B. Mode up the CBCT, click 3D3D Match, then acquire the CBCT. Zoom up the center area. Perform manual matching to place the central BB and four peripheral BBs in CBCT into the contours of BBs in CT. Record the discrepancies in cm.
C. Move the couch by 0.5 cm, 1 cm, and 2 cm in vertical, longitudinal, and lateral directions, respectively. Record the couch position.
D. Mode up the CBCT, click 3D3D Match, then acquire the CBCT. Zoom up the center area. Perform manual matching to place the central BB and four peripheral BBs in CBCT into the contours of BBs in CT. Record the shifts in cm.
E. Apply the shifts and enable motion the couch in the clinac console. Record the couch position.
F. Retract the KVD/KVS. Go into the room and adjust the couch so that the marker block phantom is set at the isocenter as initially set in “A”. Record the couch position.

**Table:**

	*Vert. (cm)*	*Lng. (cm)*	*Lat. (cm)*	*Note*
A. Initial couch position				
B. discrepancies				
C. Shifted position				
(1) = A – C: expected shift				
D. Shifts from matching				
(2) = D – (1): matching accuracy				
E. New couch position				
F. Final couch position				
(3) = D – F: couch motion accuracy				
Tolerance for B, (2) and (3):				
≤2 mm for non‐SRS/SBRT				
≤1 mm for SRS/SBRT				

**Pass/Fail:**_____

## I14–I18. CBCT Image Quality Tests Performed with a Catphan using a selected Mode

Mode:_____

Illustration of Catphan 504 Phantom (The Phantom Laboratory, NY, USA)

## I14. Geometric distortion

Date:_____Initial:_____

### a. Slice thickness (CTP 404)

Zoom in at the wire. Pick one end of the wire with one end of the measure tool, and slice up (or down) and pick the same end of the same wire with the other end of the measure tool. Slice thickness (mm)=measured discance * 0.42.

Tolerance: 0.5 mm.

**Table:**

#	*Measured Thickness*
1	
2	
3	
4	

**Pass/Fail:**_____

### b. Spatial linearity of pixel size (CTP 404)

Date:_____Initial:_____

Measure the distance between two rods.

Tolerance: 1.0 mm.

**Table:**

#	*Measured Distance*
1	
2	
3	
4	

**Pass/Fail:**_____

## I15. spatial Resolution (high contrast Resolution) (CTP 528 – spatial resolution)

Date:_____Initial:_____

Pick the least discernible bar group.

Tolerance: group6.

Discernible group:________________group

**Pass/Fail:**_____

## I16. contrast (low contrast Resolution) (CTP 515 – sensitivity or contrast test)

Date:_____Initial:_____

Count the number of discernible disks on the upper right side.

Tolerance: disk4 in supraslice 1.0% group.

Number of discernible disks:disks

**Pass/Fail:**_____

## I17. HU constancy (Hounsfield unit verification) (CTP 404)

Date:_____Initial:_____

Measure the HU value in each material disk with the standard deviation.

Tolerance: ±40  HU

**Table:**

*Material*	*HU #*	*Meas.*	*Material*	*HU #*	*Meas.*
Air	−1000		Polystyrene	−35	
Teflon	+990		LDPE	−100	
Delin	+340		PMP	−200	
Acrylic	+120				

**Pass/Fail:**_____

## I18. uniformity and Noise (CTP486)

### a. Uniformity

Date:_____Initial:_____

Select 5 cm by 5 cm ROI and measure HU in five locations.

Tolerance: ±40  HU.

**Table:**

*ROI #*	*HU #*
1	
2	
3	
4	
5	

### b. Noise

Date:_____Initial:_____

Select max. ROI and measure standard deviation of HU.

Tolerance: ±40  HU.

**Table:**

*ROI Size*	*Standard Deviation of HU #*
$10\times 10\;{\text{cm}}^{2}$	

**Pass/Fail:**_____

## I19. CBCT Image dose (Annual QA only)

Date:_____ Initial:_____

Devices: CTDI chamber, bowtie filters for full‐ and half‐fan modes and cylindrical phantoms.

Setup condition: CTDI chamber is inserted at the center hole of the cylindrical phantom where the isocenter lies at the center of the phantom. The longest dimension of the CTDI chamber is aligned along with H‐F laser or crosshair, which can be accomplished by aligning the phantom with the corresponding laser.

**Table:**

*CBCT Protocols*	*Phantom*	*Integrated Dose‐length Value Baseline (mGy.cm)*	*Year*
Standard‐Dose Head	Small		
Low‐Dose Head	Small		
High‐Quality Head	Small		
Pelvis Large			
Pelvis Spot Light	Large		
Low‐Dose Thorax	Large		

**Pass/Fail:**_____
